# The therapeutic potential of atorvastatin in treatment of *G. lamblia* infected mice; *in silico*, parasitological, and histopathological study

**DOI:** 10.3389/fvets.2026.1788316

**Published:** 2026-06-02

**Authors:** Khalil Mohamed, Marawan Khodary, Dina hamed, Abdullah Alhazmi, Hattan S. Gattan, Mohammed H. Alruhaili, Eman Fathi Fadel, Hatem A. Elshabrawy, Asmaa M. El-kady

**Affiliations:** 1 Department of Epidemiology and Medical Statistics, Faculty of Public Health and Health Informatics, Umm Al-Qura University, Mecca, Saudi Arabia; 2 Faculty of Medicine, Qena University, Qena, Egypt; 3 Department of Medical Parasitology, Faculty of Medicine, Qena University, Qena, Egypt; 4 Department of Medical Laboratory Sciences, Faculty of Applied Medical Sciences, King Abdulaziz University, Jeddah, Saudi Arabia; 5 Special Infectious Agents Unit, King Fahad Medical Research Center, King AbdulAziz University, Jeddah, Saudi Arabia; 6 Department of Clinical Microbiology and Immunology, Faculty of Medicine, King AbdulAziz University, Jeddah, Saudi Arabia; 7 Department of Medical Parasitology, Faculty of Medicine, Sohag University, Sohag, Egypt; 8 Department of Molecular and Cellular Biology, College of Osteopathic Medicine, Sam Houston State University, Conroe, TX, United States

**Keywords:** giardiasis, *in silico*, *in vivo*, metronidazole, statins

## Abstract

**Background:**

*Giardia lamblia* infection (giardiasis) remains a significant global health concern, and the emergence of drug resistance to the first-line treatment, Metronidazole (MTZ), necessitates the exploration of alternative therapeutic agents. This study investigates the potential of Atorvastatin, as a repurposed drug for giardiasis, focusing on its direct antiparasitic activity and its role in modulating the associated inflammatory pathology.

**Methods:**

Molecular docking simulations were performed to assess the binding affinity of Atorvastatin and MTZ against four key enzymes in the *G. lamblia* mevalonate pathway, which are farnesyl transferase (ftase), isopentenyl pyrophosphate isomerase (ipp), mevalonate diphosphate decarboxylase (mvd), and mevalonate kinase (mvk). An *in vivo* experimental model utilized Swiss albino mice infected with *G. lamblia* and treated with MTZ (120 mg/kg/day for 7 days) or Atorvastatin (20 mg/kg/day and 40 mg/kg/day for 5 days) was used. Efficacy of treatments was evaluated by quantifying intestinal trophozoite counts. Furthermore, the study assessed liver function (ALT/AST), intestinal and liver histopathology, and the expression of the inflammatory markers inducible Nitric Oxide Synthase (iNOS) and interleukin 6 (IL 6) in the intestinal tissues.

**Results:**

Molecular docking revealed that Atorvastatin exhibited comparable or slightly superior binding affinities to the targeted *G. lamblia* enzymes compared to MTZ, with the strongest interaction observed with ipp_v6th89 (−5.7 kcal/mol). *In vivo*, Atorvastatin demonstrated significant, dose-dependent antiparasitic activity, achieving a 41.54% reduction in trophozoite counts at 40 mg/kg, though this was lower than the 71.73% reduction achieved by MTZ. Crucially, Atorvastatin exhibited a superior anti-inflammatory effect. The higher dose of Atorvastatin suppressed iNOS expression to 5.40% of the reaction area, a nearly four-fold reduction compared to the infected untreated group (19.59%) and significantly better than the modest reduction seen with MTZ (17.49%). On the other hand, Atorvastatin (at a dose of 40 mg /kg) proved to be the most potent intervention, suppressing IL-6 expression to a low average of 6.12% in comparison to 26.7 and 17.6% reaction area for infected untreated and MTZ treated mice, respectively. Histopathological analysis confirmed that Atorvastatin treatment effectively restored intestinal architecture and mitigated liver pathology, correlating with a significant reduction in elevated serum ALT levels.

**Conclusion:**

Atorvastatin possesses a dual therapeutic role against giardiasis. It acts as a moderate antiparasitic agent, likely by targeting the mevalonate pathway, and as a potent immunomodulatory agent. Its superior ability to suppress the inflammatory response and mitigate intestinal and hepatic pathology suggests that Atorvastatin is a promising candidate for an adjunct therapy to Metronidazole, particularly in cases where inflammation and chronic sequelae are major concerns.

## 1 Introduction

Human giardiasis, caused by the protozoan parasite *Giardia lamblia* (syn. *Giardia duodenalis*, *Giardia intestinalis*), is one of the most prevalent enteric protozoan infections globally, with prevalence rates ranging from 2 and 5% in the developed world and 20–30% in the developing countries (1). The World Health Organization (WHO) has included giardiasis in its Neglected Disease Initiative (NDI) since 2004 owing to its establishment in developing countries, in addition to the general lack of information behind the molecular mechanism of the disease (2, 3). Egypt is classified as a low-to middle-income nation, where *Giardia* is a prevalent pathogen, with prevalence ranging from 21 to 50% (4).

*Giardia lamblia* infection occurs through the ingestion of cysts present in contaminated water and food or by direct person–person contact. The cyst exhibits resistance to environmental conditions and can persist for prolonged durations in cool, moist environments, promoting the spread of giardiasis (4). Central to its pathogenicity is the parasite’s ability to attach to the intestinal epithelium and affect the host’s immune system, allowing it to evade detection and sustain infection. *G. lamblia* employs several virulence mechanisms, including adhesion to the intestinal epithelium, induction of apoptosis in enterocytes, secretion of proteases, and reduction in nutrient absorption and secretion (5). Clinically, giardiasis can range from an asymptomatic carrier state to severe illness, manifested as diarrhoea, dehydration, abdominal pain, malabsorption, steatorrhea and weight loss, often lasting for several weeks or months in untreated children (6).

Metronidazole (MTZ) is the standard drug used for giardiasis but unfortunately, it has many adverse effects as nausea, metallic taste, hepatic damage, and possible carcinogenicity along with progressive widespread resistance to this drug. Consequently, searching for more effective, natural, and safe alternative drugs is a priority to overcome these problems (7). Currently, drug repurposing is a key approach in drug development, and the use of medications (approved by the FDA) with known safety profiles offers results in less time (63).

One of the major undisputed clinical breakthroughs of the 20th century was the discovery of the statin family of drugs (8). These compounds are renowned for their ability to lower cholesterol levels and are used to treat approximately 40 million individuals with high cholesterol levels worldwide (9). Their mechanism of action consists of promoting the reduction of cholesterol synthesis in hepatocytes by the inhibition of the enzyme hydroxymethyl-glutaryl-CoA (HMGCoA) reductase, preventing the transformation of HMG-CoA into mevalonic acid and, consequently, the cholesterol biosynthesis (10, 11). Although their primary mechanism relates to lipid metabolism in humans, the underlying biochemical pathway it targets, the mevalonate pathway, is also present and essential in various organisms, including some protozoan parasites. The present study aimed to evaluate the therapeutic potential of Atorvastatin in the context of giardiasis, examining the theoretical mechanism of action and the limited preclinical and clinical evidence (12). Evidence supports the idea that statins play an anti- parasitic role, partly owing to their ability to reduce cholesterol, increase phagocytosis and produce anti- parasitic molecules. They were used for the experimental management of parasitic infections such as Chagas disease, leishmaniasis, toxoplasmosis, or malaria (13).

The rationale for considering Atorvastatin, an HMG-CoA reductase inhibitor, against *G. lamblia* stems from the parasite’s reliance on the mevalonate pathway for synthesizing essential isoprenoids. Isoprenoids are a diverse class of molecules vital for numerous cellular functions, including the post-translational modification of proteins known as isoprenylation. This process involves the covalent attachment of isoprenoid lipids, such as farnesyl pyrophosphate (FPP) and geranylgeranyl pyrophosphate (GGPP), to specific target proteins, often influencing their localization, interactions, and activity (14).

The present study aimed to evaluate the therapeutic potential of Atorvastatin in the context of giardiasis, examining the theoretical mechanism of action and the limited preclinical and clinical evidence (12). Evidence supports the idea that statins play an anti- parasitic role, partly owing to their ability to reduce cholesterol, increase phagocytosis and produce anti- parasitic molecules. They were used for the experimental management of parasitic infections such as Chagas disease, cryptosporidiosis, leishmaniasis, toxoplasmosis, or malaria (15–18). While statins have demonstrated antiparasitic activity against protozoa through HMG-CoA reductase inhibition, their potential against *Giardia* remains entirely unexplored. The present study fills this gap by evaluating atorvastatin’s efficacy in a murine Giardia model, aiming to establish proof-of-concept for repurposed statins in anti-giardial therapy compared to metronidazole and untreated controls, via parasite burden, histopathology, and clinical outcomes.

## 2 Materials and methods

### 2.1 Ethical consideration

This research was carried out at the animal facility and the Medical Parasitology Department of the Faculty of Medicine at South Valley University in Qena, Egypt. All experimental procedures adhered to the guidelines established by the Animal Care and Use Committee at the Faculty of Medicine, South Valley University, Qena, Egypt. Furthermore, the design of the study received approval from the Institutional Research Committee at the same faculty (SVU- MED- PAR008-4-25-1-19).

### 2.2 Docking analysis

#### 2.2.1 Ligand preparation

The ligands used upon this study Metronidazole and Statin were retrieved from PubChem then Energy minimization was performed using the MMFF94 (19) force field implemented in Avogadro 1.2.0 (20) software to obtain optimized conformations of the ligands.

#### 2.2.2 Protein preparation

The structure of the farnesyl transferase (ftase), isopentenyl pyrophosphate isomerase (ipp), mevalonate diphosphate decarboxylase (mvd), and mevalonate kinase (mvk) for *G. lamblia* (strain ATCC 50803/WB clone C6) proteins were retrieved from the UniProt database and missing structures were generated using alphafold server (21). The binding sites for these proteins were predicted based on literature information and validated using the CB-DOCK2 (22). The proteins were prepared for docking using AutoDock Tools 1.5.7 (23), which involved removing water molecules, adding polar hydrogens, and assigning Gasteiger charges.

#### 2.2.3 Active site prediction and molecular docking

The binding sites for each protein were predicted using the CB-Dock2 server (22), which implements a cavity detection algorithm to identify potential binding pockets. Molecular docking simulations were performed using QuickVina-2 (QVina2) (24) software to evaluate the binding interactions.

### 2.3 Animals

Fifty male Swiss albino mice each between 3 to 4 weeks old and weighing between 20 and 25 grams were obtained from the animal facility at the Theodore Bilharz Research Institute (TBRI) in Giza, Egypt. These mice were raised in a controlled environment that adhered to specified pathogen-free standards. Prior to the commencement of the experiments, stool samples were collected and analyzed over a period of three consecutive days to confirm the absence of intestinal parasites in the mice.

### 2.4 Parasite preparation and induction of infection

Fresh stool specimens were collected from patients infected with *G. lamblia* after an informed consent was taken from them or from guardians (if patients are less than 18). Samples were free from other parasitic infections. All stool specimens underwent immediate processing in the parasitology laboratory. To prepare the samples, emulsification in saline was performed, followed by sieving to eliminate larger particles. The cysts were then concentrated through repeated centrifugation at 2000 rpm for 5 min, along with washing in saline. Following the final wash, the sediment was thoroughly mixed with normal saline, and the concentration of cysts in the resulting suspension was adjusted to 10,000 cysts/ml using a haemocytometer (25), which is the dose required for infection of each mouse (26–28). To verify the establishment of infection, daily assessments of the stool samples from the animals were conducted to detect the presence of *G. lamblia* cysts using iodine-stained smears. Stool samples were collected sequentially from each mouse in a dry, labeled, wide-mouth plastic container equipped with a secure lid, ensuring immediate examination through either direct iodine-stained smears or a concentration method.

### 2.5 Drug preparation and dose adjustment

- Atorvastatin 20 mg tablets were utilized in this study. The tablets were crushed and freshly dissolved in distilled water, then administered orally at dosages of 20 and 40 mg/kg/day over a period of five consecutive days (16, 29).
- Metronidazole: Metronidazole (Flagyl) tablets 500 mg (Sanofi Aventis) were used in this study. The tablets were crushed and freshly dissolved in distilled water, then administered orally at dosages of 120 mg/kg/ day for seven consecutive days (30, 31).

Treatment involving Atorvastatin and Metronidazole started on the sixth day following infection (dpi), coinciding with the peak of intestinal colonization (32). The administration of the treatments was facilitated using a stainless steel oesophageal tube.

Fifty mice were categorized into five distinct groups, each consisting of ten mice.

Group 1: Non-infected-non treated (negative control).

Group 2: Infected-non treated (positive control).

Group 3: Infected and treated with MTZ orally at a dose of 120 mg/kg/day for 7 consecutive days.

Group 4: Infected and treated with Atorvastatin orally at dosages of 20 mg/kg/day five consecutive days (16, 29).

Group 5: Infected and treated with Atorvastatin orally at dosages of 40 mg/kg/day five consecutive days (16, 29).

All mice were anesthetized with isoflurane by the inhalation route and euthanized by cervical dislocation three days after completion of the respective treatment courses (25, 31), and tissues, specifically the small intestine and livers, were harvested for the assessment of drug efficacy.

### 2.6 Evaluation of the effectiveness of the extract

#### 2.6.1 Quantification of trophozoites in various groups of infected mice

Following the euthanasia of the mice, the small intestine was extracted, and the contents of the duodenum underwent a parasitological analysis to determine the quantity of *G. lamblia* trophozoites. The *G. lamblia* trophozoites were then counted microscopically across 10 fields using a 100x objective lens (28). The percent reduction (PR) was calculated using a specific formula, indicating the decrease in trophozoites numbers in the treated group relative to the infected untreated group across five consecutive fields per animal (31, 33).

$$\text{Percent Reduction}\;(\mathrm{PR})=\frac{\begin{array}{l} \text{mean trophozoites count in control group}\\ –\text{mean trophozoites count in treated group} \end{array}}{\text{mean trophozoites count in control group}}\;x\;100$$

#### 2.6.2 Measurement of liver enzymes

Blood samples were obtained from mice that were sacrificed. Serum samples were analysed to assess liver enzymes (AST and ALT) utilizing commercial kits from Biodiagnostic Co., Birmingham, UK.

#### 2.6.3 Histopathological examination

Five micrometers sections were prepared from Formalin-fixed paraffin-embedded intestinal and liver tissue blocks. The process involved deparaffinizing the sections in xylene, followed by rehydration through a series of decreasing alcohol concentrations (100, 80, 70, and 50%; each for 1 min), and rinsing in distilled water, which was subsequently followed by a wash in running tap water for 3 to 5 min. The slides underwent staining with haematoxylin for 5 to 7 min, were then washed in running tap water, and subsequently stained with eosin for 3 to 5 min before another rinse in running tap water. To complete the preparation, the slides were dehydrated in ascending concentrations of ethanol (50, 70, 80, and 100%; each for 1 min), cleared in xylene, and mounted using dibutyl phthalate polystyrene xylene (DPX) before being covered with a slip.

#### 2.6.4 Immunohistochemical study

Immunohistochemical examination of IL-6 and iNOS was performed using paraffin-embedded tissue sections, each with a thickness of 5 μm. Following dewaxing, dehydration, and treatment with 2% hydrogen peroxide, the sections were incubated with normal goat serum for 30 min and subsequently overnight with primary antibodies: anti-IL-6 (Bimake, Shanghai, China; diluted 1:100) and anti-iNOS (Santa-Cruz Biotechnology Inc., Burlingame, CA; diluted 1:100). The sections were rinsed with 20 mM phosphate-buffered saline (pH 7.3) and incubated for 30 min with biotinylated anti-rabbit secondary antibodies at a dilution of 1:200, followed by another rinse. The sections were treated for 30 min with the avidin-biotin peroxidase complex and then exposed to 3,3′-diaminobenzidine for 10 min. Afterward, the sections were counterstained with hematoxylin. Negative control slides were prepared from the same tissue block, excluding the primary antibody to evaluate non-specific binding.

Scoring of the immunohistochemistry results was conducted by determining the reaction area percentage in 10 microscopic fields using Image J 1.53 t, developed by Wayne Rasband and contributors at the National Institutes of Health, USA. Positivity was evaluated through a thorough examination of all slides at a lower magnification (40×), followed by the random selection of ten distinct fields at a higher magnification (400×) to ascertain the mean percentage of cells exhibiting immunolabeling. Multiple regions were analyzed to improve the reproducibility of the findings. Stained sections were classified as positive if more than 0% of the cells showed immunostaining for iNOS and IL-6. The immunohistochemically stained sections were visualized using an Olympus microscope (BX-53). The automated quantitative evaluation of marker expression was introduced to address the recognized shortcomings of manual scoring concerning reproducibility (34, 35). Image analysis was utilized to assess the selected foci based on an automatic evaluation of color staining intensity and reaction area, resulting in a percentage area score. The immunohistochemically stained slides were assessed independently by two separate pathologists using the threshold method, and the average of the scores from both pathologists across 10 high-power fields per slide was documented.

### 2.7 Statistical analysis

The data were subjected to analysis using the Statistical Package for the Social Sciences (SPSS) version 26 (IBM Corp., Armonk, NY, USA), presented as mean ± SD. Group comparisons were performed using two-way analysis of variance (ANOVA) accompanied by Tukey *post hoc* tests. A *p*-value of less than 0.05 was considered statistically significant.

## 3 Results

### 3.1 Docking results

**Figure 1:**
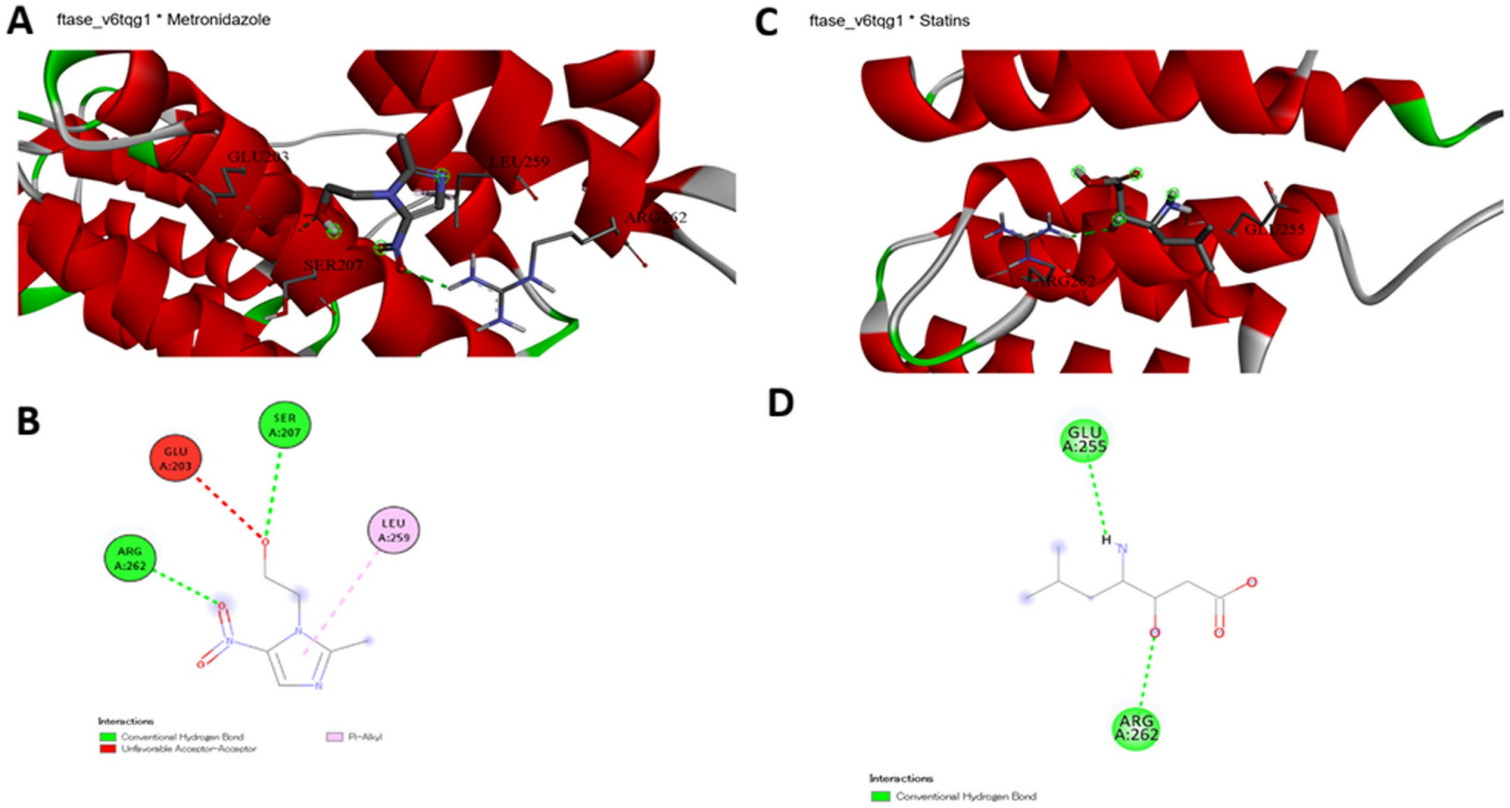
3D (upper panel) and 2D (lower panel) ligand-interacted forms show key amino acid residues of ftase_v6tqg1 with Metronidazole **(A,B)** and Atorvastatin **(C,D)** using BIOVIA drug discovery studio visualizer.

**Figure 2:**
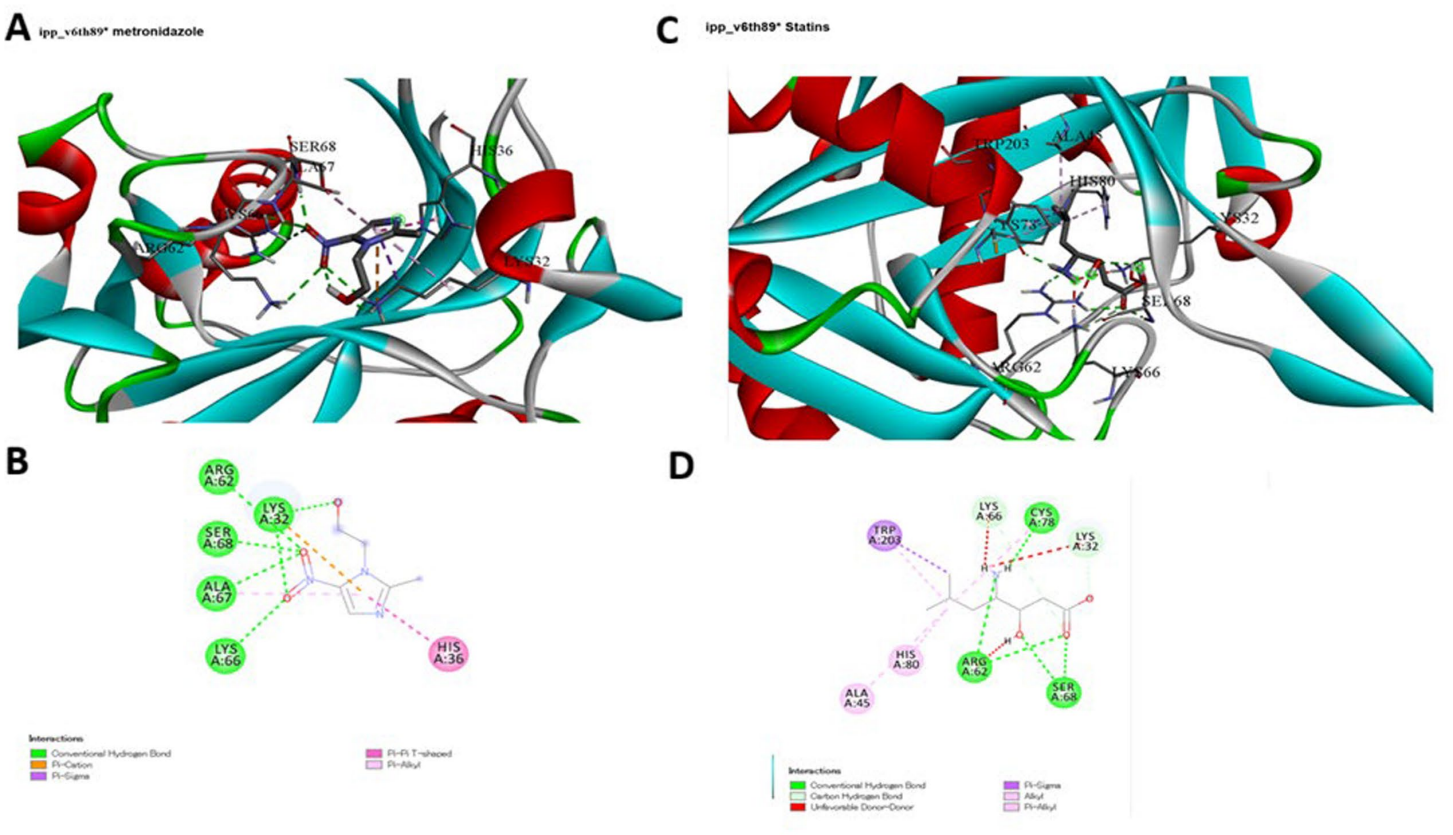
3D (upper panel) and 2D (lower panel) ligand-interacted forms show key amino acid residues of ipp_v6th89 with Metronidazole **(A,B)** and Atorvastatin **(C,D)** using BIOVIA drug discovery studio visualizer.

**Figure 3:**
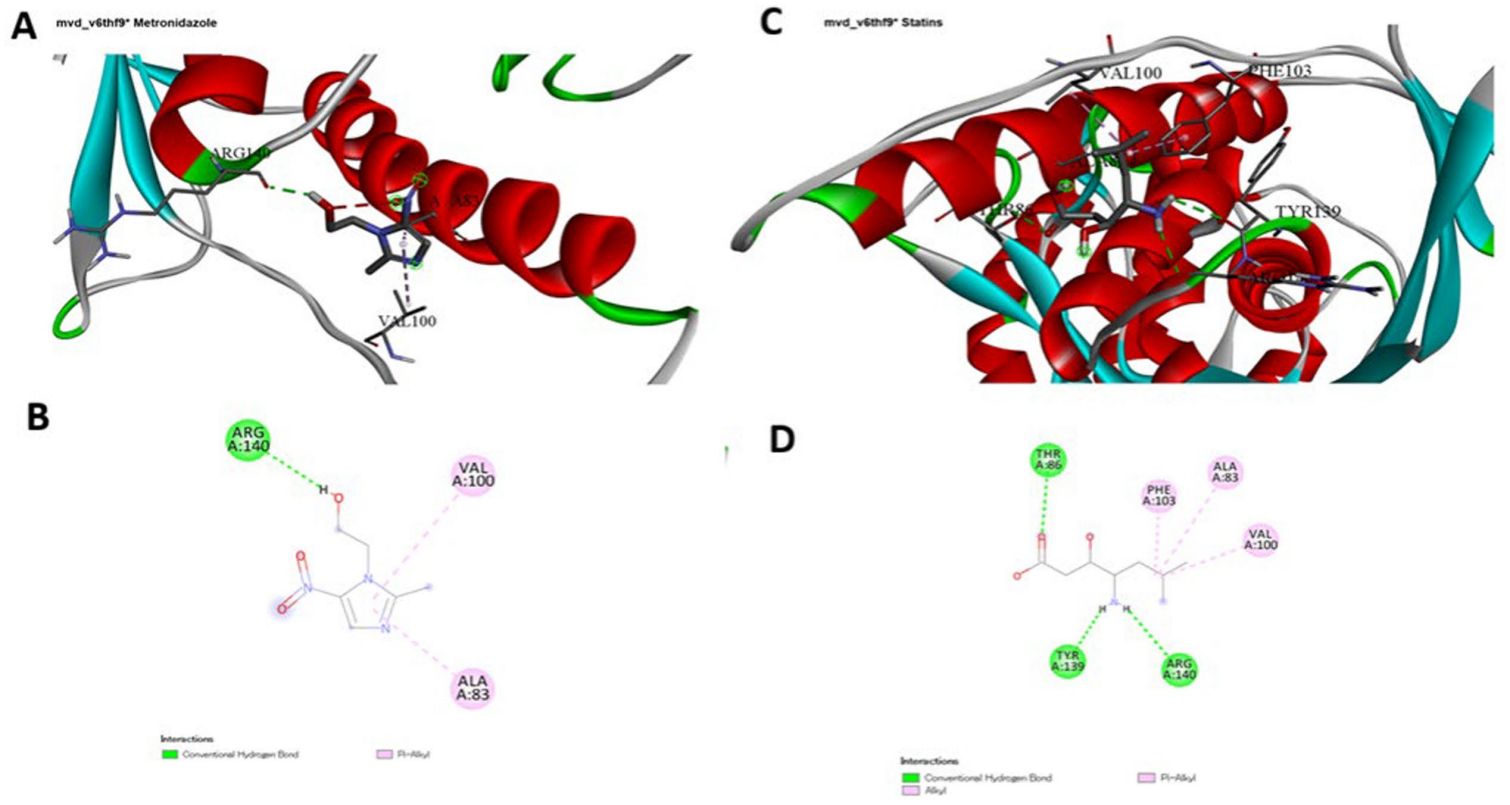
3D (Upper panel) and 2D (lower panel) ligand-interacted forms show key amino acid residues of mvd_v6thf9 with metronidazole **(A,B)** and atorvastatin **(C,D)** using BIOVIA drug discovery studio visualizer.

**Figure 4:**
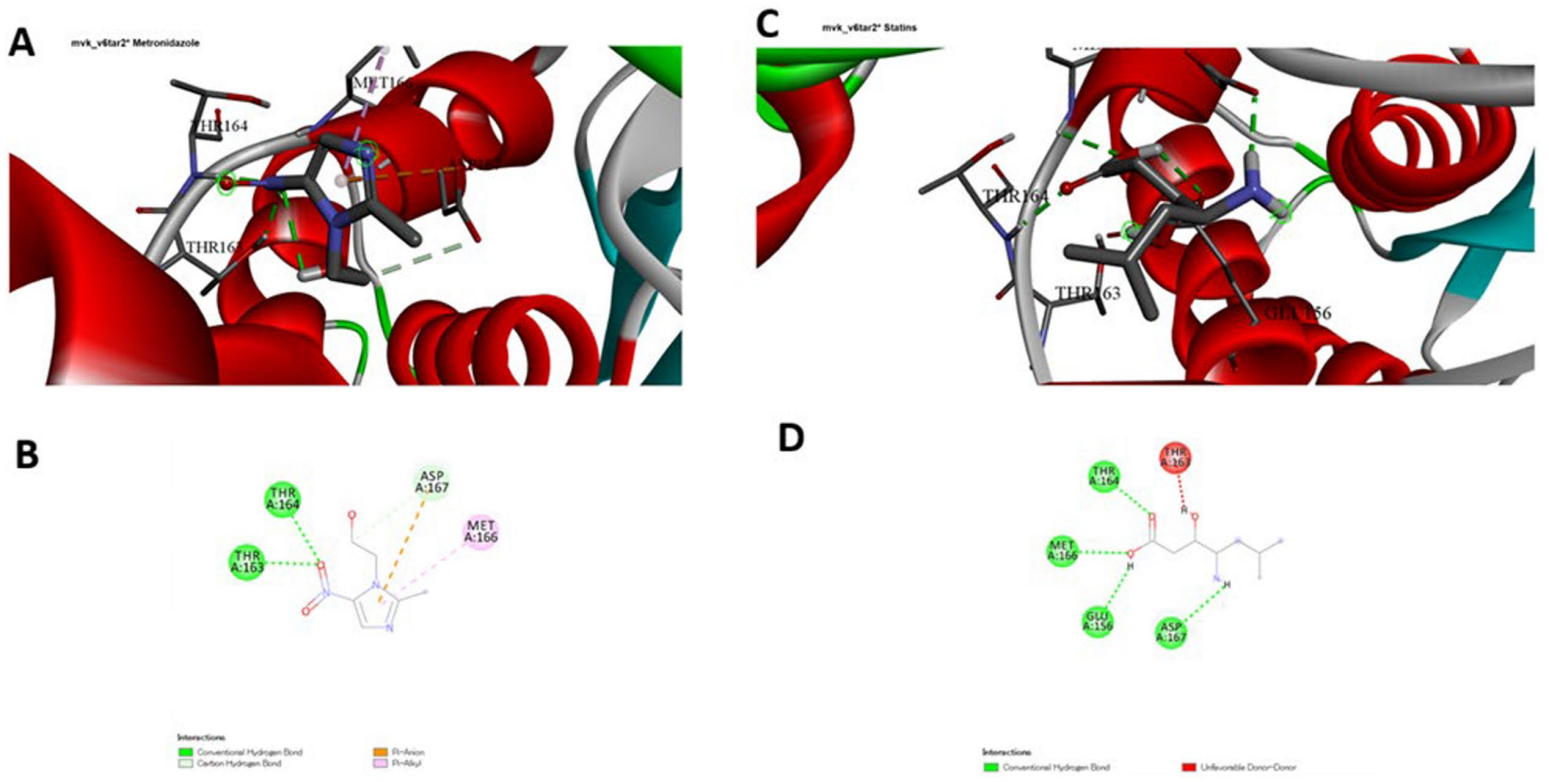
3D (upper panel) and 2D (lower panel) ligand-interacted forms show key amino acid residues of mvk_v6tar2 with metronidazole **(A,B)** and atorvastatin **(C,D)** using BIOVIA drug discovery studio visualizer.

Atorvastatin and metronidazole demonstrated largely similar docking scores against the four Giardia enzymes, with Atorvastatin typically exhibiting slightly more favorable (more negative) binding energies, especially for IPP isomerase (−5.7 vs. −4.9 kcal/mol) and MVD. In contrast, Metronidazole frequently established more extensive hydrogen bonding and aromatic interaction networks in certain targets, Figures 1–4 and Tables 1, 2.

**Table 1:** Showing the dimensions (x, y, z) and center of the search cube.

Receptor	center_x	center_y	center_z	size_x	size_y	size_z
fold_ftase_v6tqg1_model_0.pdb	23	8	−11	30	30	30
fold_ipp_v6th89_model_0.pdb	−7	0	5	30	30	30
fold_mvd_v6thf9_model_0.pdb	−13	−24	−6	30	30	30
fold_mvk_v6tar2_model_0.pdb	−10	2	−7	30	30	30

**Table 2:** Showing ligands RMSD (Root-Mean-Square Deviation) for metronidazole and statins against the tested enzymes.

Protein	Ligand	Average RMSD lb
Ftase	Metronidazole	1.888
Ftase	Statins	1.836
Ipp	Metronidazole	2.165
Ipp	Statins	3.113
Mvd	Metronidazole	2.602
Mvd	Statins	2.878
Mvk	Metronidazole	2.384
Mvk	Statins	2.524

For FTase, Atorvastatin docked with an estimated binding free energy of around −4.5 kcal/mol, closely resembling metronidazole (−4.4 kcal/mol), which suggests a comparable predicted affinity for this target. Atorvastatin formed two conventional hydrogen bonds with ARG262 and GLU255 at short bond distances of 2.33 and 2.47 Å, respectively. Metronidazole also created two conventional hydrogen bonds, involving SER207 and ARG262, with bond lengths ranging from 2.28 to 2.99 Å, in addition to a longer contact of approximately 5.01 Å. While Atorvastatin did not report any hydrophobic contacts with FTase, Metronidazole engaged in an additional Pi alkyl hydrophobic interaction with LEU259, indicating a mixed binding mode of hydrogen bonds and hydrophobic interactions despite the nearly identical docking energies.

In the case of IPP isomerase, Atorvastatin exhibited a more advantageous binding energy of (−5.7 kcal/mol) compared to metronidazole (−4.9 kcal/mol), indicating a more complex formation. Atorvastatin formed five conventional hydrogen bonds along with two carbon hydrogen bonds, primarily with residues such as Val114, Asn113, and Asp377, with the majority of bond distances ranging from approximately 2.07 to 2.83 Å, and several additional longer contacts reaching up to ~5 Å. Conversely, metronidazole established an even denser hydrogen-bonding network, consisting of eight conventional hydrogen bonds involving various Lys and Arg residues (for instance, Lys32, Arg62, Lys66, Ala67, Ser68), with distances predominantly within the 2.0–3.0 Å range. Furthermore, Metronidazole engaged in several aromatic and electrostatic interactions, including Pi–Pi T shaped Pi alkyl, Pi sigma, and Pi cation contacts, demonstrating tight and multifunctional binding despite its slightly less favorable docking score.

For MVD, Atorvastatin exhibited a more advantageous energy of (−4.8 kcal/mol) compared to metronidazole, which had an energy of (−4.2 kcal/mol). Atorvastatin established several conventional hydrogen bonds with THR86, ARG140, and TYR139, with bond distances primarily ranging from 2.56 to 3.04 Å, along with additional contacts around 4.2 to 4.7 Å. These polar interactions were enhanced by alkyl and Pi alkyl hydrophobic interactions with ALA83, VAL100, and PHE103, suggesting a synergistic polar and hydrophobic stabilization of the complex. Conversely, metronidazole formed only a single conventional hydrogen bond with ARG140 (bond lengths varying from 2.22 to 5.27 Å) and two Pi alkyl interactions with ALA83 and VAL100, leading to a less complex interaction pattern that aligns with its lower binding energy.

For MVK, the binding energies of Atorvastatin and metronidazole were quite similar, at around −5.1 and −5.0 kcal/mol, respectively. Atorvastatin established four conventional hydrogen bonds with THR164, MET166, ASP167, and GLU156, exhibiting short bond lengths of approximately 2.0–2.6 Å, which suggests a dense polar interaction pattern; no further hydrophobic or electrostatic bonds were recognized. Metronidazole formed three conventional hydrogen bonds along with one carbon hydrogen bond involving THR163, THR164, and ASP167, with similar bond distances in the range of 2.0–2.7 Å, and participated in a Pi alkyl interaction with MET166 and a Pi anion interaction with ASP167. This combination of hydrogen bonding and electrostatic contacts results in a binding mode that is fundamentally comparable in strength to that of Atorvastatin for this enzyme (Figures 1–4).

#### 3.1.1 Statins decreased trophozoite levels in *G. lamblia*-infected subjects

**Figure 5:**
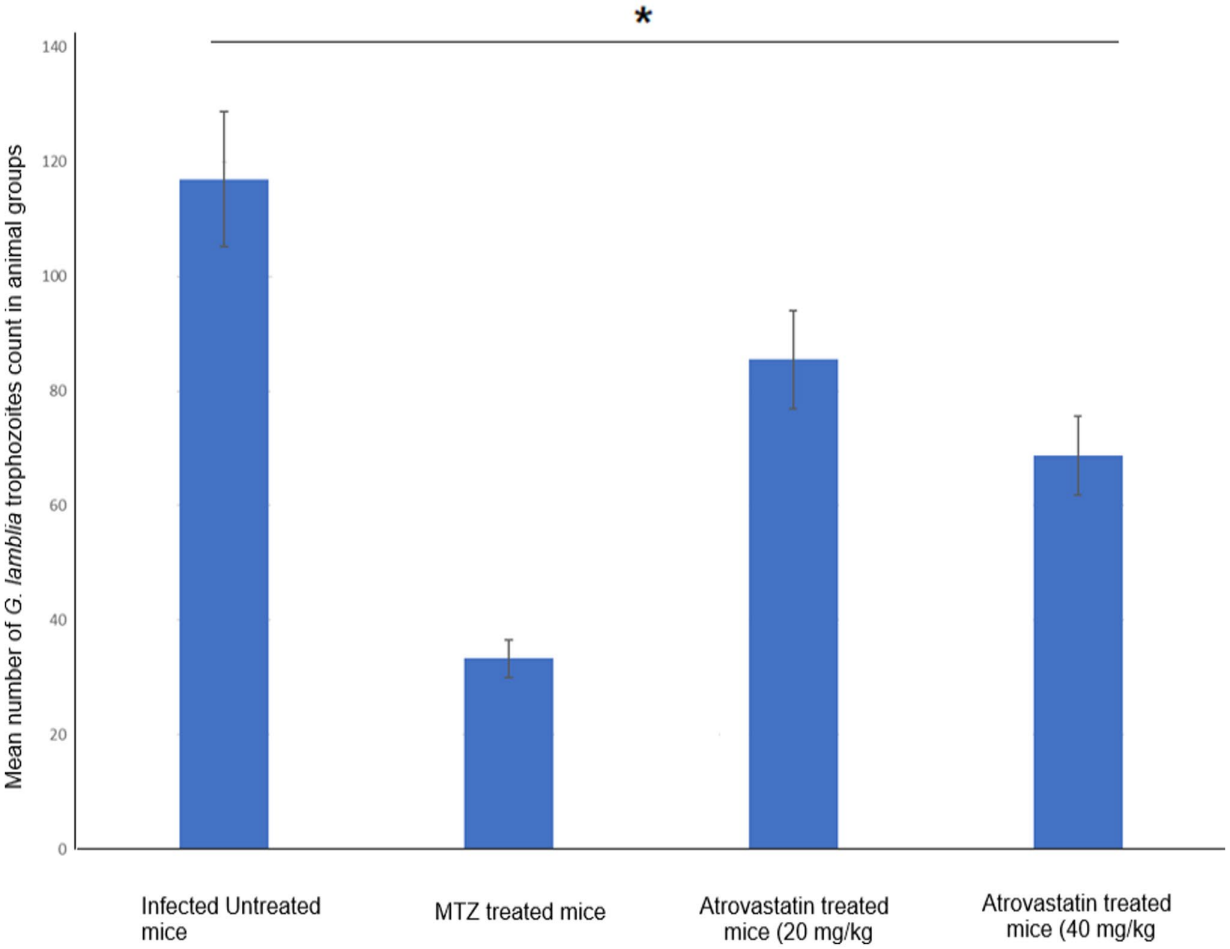
Effect of Atorvastatin or Metronidazole treatments on the number of *G. lamblia* trophozoite. Data are expressed as means (*n* = 10) with error bars representing SD and were analysed using ANOVA and Tukey as a *post hoc* test. Asterisks (*) indicate a significant difference in the numbers of trophozoite in treated groups compared to the infected untreated group (*p* = 0.003), and a significant difference between the Metronidazole- and statins- treated groups, *p* = 0.001.

Examination of the small intestines across all animal groups revealed a significant variation in trophozoite counts among treated animals when juxtaposed with infected untreated mice (*p* = 0.001). The animals that were administered Metronidazole and Atorvastatin exhibited a notable reduction in trophozoite counts compared to the infected untreated group. Reductions of 71.73, 41.54, and 27.30% for Metronidazole, Atorvastatin at a dosage of 40 mg/kg, and Atorvastatin at a dosage of 20 mg/kg, respectively were reported (*p* = 0.001) for Metronidazole, *p* = 0.002 for Atorvastatin at 40 mg/kg, and *p* = 0.004 for Atorvastatin at 20 mg/kg in relation to infected untreated animals (Figure 5 and Table 3).

**Table 3:** Showing treatment with atorvastatin reduced oocysts counts in the stool of infected mice.

Animal group	Oocyst count/HPF Mean ± SD	*%R*	*p-*value (among groups)	Post hock test
Infected Untreated	117.6 + 6.22		0.002*	
Infected + MTZ	33.25 + 5.56	71.73%		a, b, c
Infected + Atrovastatin (20 mg/kg)	85.5 + 4.93	27.30%		d
Infected + Atrovastatin (40 mg/kg)	68.75 + 3.5	41.54%		

Data are expressed as mean ± SD and were analysed using ANOVA for pairwise comparison. % R: percentage of reduction. SD: Standard deviation. Letter “a” indicates significant reductions in oocysts counts after treatments compared to infected untreated group. Letter “b” indicates a significant difference in oocysts versus MTZ- treated group. Letter “c” indicates a significant difference in oocysts count versus Atrovastatin (20 mg/kg) treated group. Letter “d” indicates a significant difference in oocysts count versus Atrovastatin (40 mg/kg) treated group. Asterisk (*) indicates significant difference between all groups.

#### 3.1.2 Statins restored normal intestinal pathology

**Figure 6:**
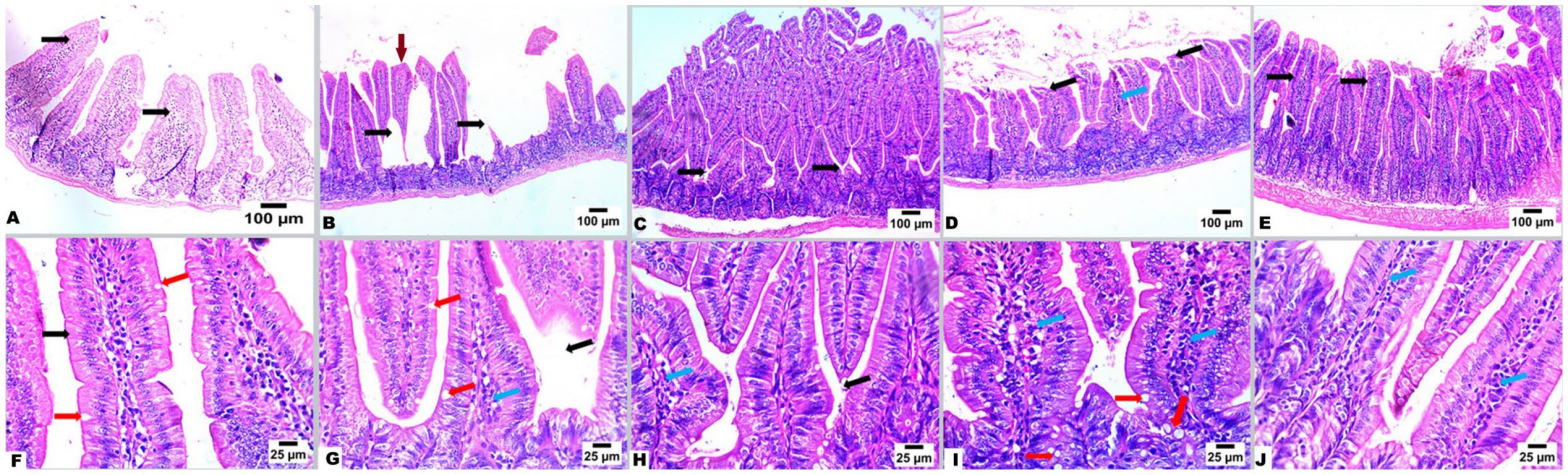
Photomicrographs showing sections of the small intestine of mice groups stained by H and E. **(A–E)** Intestinal sections magnification power 100x. **(A)** Sections in the small intestine of normal control mice showing regular villous pattern (black arrows) (100x). **(B)** Sections in the small intestine of infected untreated control mice showing short blunted villi (red arrow) with desquamation of the villi (black arrows). **(C)** Sections in the small intestine of MTZ treated mice showing desquamated villi (black arrows) **(D)** Sections in the small intestine of Ator-20 treated mice showing villous blunting (black arrows) with mild cellular infiltration (blue arrow). **(E)** Sections in the small intestine of Ator-40 treated mice showing mostly regular villous pattern (black arrows). **(F–J)** Intestinal sections magnification power 400x. **(F)** Sections in the small intestine of normal control mice showing regular villous pattern (black arrows) and few distributed goblet cells (red arrows). **(G)** Sections in the small intestine of infected untreated control mice showing desquamated villi (black arrow), few scattered goblet cells (red arrows) and infiltration of the villi core with inflammatory cells infiltrate (blue arrows). **(H)** Sections in the small intestine of MTZ treated mice showing desquamated villi (black arrows) and inflammatory cell infiltrates in core villi (blue arrow). **(I)** Sections in the small intestine of Ator-20 treated mice showing inflammatory cell infiltrates in core villi (blue arrow) and increased number of goblet cells (red arrow). **(J)** Sections in the small intestine of Ator-40 treated mice showing mostly regular villous pattern with mild infiltration with inflammatory cells (blue arrows).

To investigate the impact of Metronidazole and Atorvastatin treatments on the small intestinal lesions caused by giardiasis, sections from the proximal small intestine were stained using hematoxylin and eosin (H&E). The small intestinal sections from uninfected animals displayed normal intestinal villi and crypts (black arrows), as indicated in Figures 6A,F. Villi showed typical structure characterized by surface simple columnar epithelial cells. The core of the villi was composed of connective tissue and normally distributed goblet cells (red arrows). In contrast, small intestinal sections from untreated infected animals exhibited significant shortening and damage to the intestinal villi (red arrows) with marked villous desquamation (black arrows), along with retraction of their connective tissue cores, as shown in Figure 6B. Higher magnification showed desquamated villi (black arrow), few scattered goblet cells (red arrows) and infiltration of the villi core with inflammatory cells infiltrate (blue arrows), (Figure 6G).

Conversely, tissue sections from animals treated with Metronidazole exhibited desquamated villi (black arrows) and inflammatory cell infiltrates in core villi (blue arrow), (Figures 6C,H). Sections in the small intestine of infected mice treated by Atrovastatin at a dose of 20 mg/kg (Figure 6D) showed villous blunting (black arrow) and inflammatory cell infiltrates in core villi (blue arrow), while Figure 6I exhibted increased number of goblet cells (red arrow) with mild cellular infiltration (blue arrow). Sections in the small intestine of infected mice treated with at a dose of 40 mg/kg (Figures 6E,J) showed mostly regular villous pattern (black arrows) and mild infiltration of the villous core by chronic inflammatory cells (blue arrows).

### 3.2 Effect of satins on *G. lamblia* induced liver pathology

**Figure 7:**
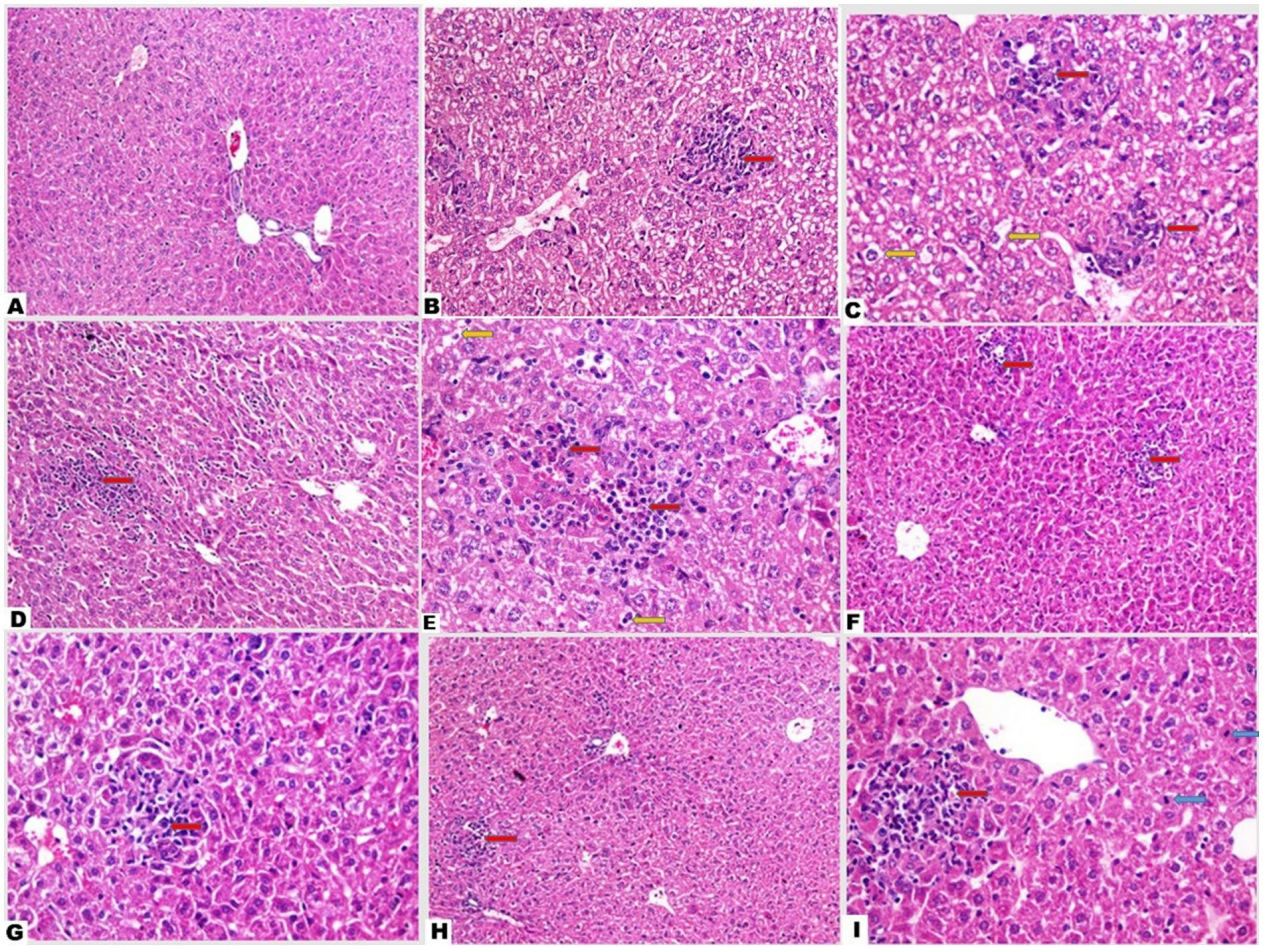
Photomicrograph showing sections of the liver tissues of mice groups stained by H and E. **(A)** Sections in the liver of normal control mice showing regular hepatic lobular pattern with no evidence of inflammatory changes. **(B,C)** Sections in the liver of infected untreated mice showing irregular liver cell cords and focal lobular infiltration by mononuclear inflammatory cells (red arrows) with hydropic changes of hepatocytes (yellow arrow). **(D,E)** Sections in the liver of infected mice treated by MTZ showing irregular liver cell cords with hydropic changes of hepatocytes (yellow arrow) and focal lobular infiltration by mononuclear inflammatory cells (red arrows). **(F,G)** Sections in the liver of infected mice treated by ATO 20 showing focal lobular infiltration by inflammatory cells (red arrows). **(H,I)** Sections in the liver of infected mice treated by ATO 40 showing focal lobular infiltration by inflammatory cells (red arrows) and few apoptotic figures (blue arrows).

While *G. lamblia* primarily affects the small intestine, it can occasionally lead to liver complications through several mecha nisms. One way is through the biliary tract, causing cholangitis or even contributes to gallstones or pancreatitis. Additionally, *G. lamblia* can induce liver inflammation, as seen in some cases of chronic hepatitis, or cause changes in liver histology, such as steatosis (fatty liver) (36–39). Therefore, we examined liver tissue sections from all mice groups. As shown in Figure 7, Sections in the liver of normal control mice showed regular hepatic lobular pattern with no evidence of inflammatory changes (Figure 7A). In contrast, the liver sections of infected untreated mice exhibited irregular liver cell cords and focal lobular infiltration by mononuclear inflammatory cells (red arrows) with cellular hydropic degeneration (yellow arrows) (Figures 7B,C). The liver sections of infected mice treated with MTZ showed irregular liver cell cords with hydropic changes of hepatocytes (yellow arrow) and focal lobular infiltration by mononuclear inflammatory cells (red arrows) (Figures 7D,E). The liver sections of infected mice treated with statins at a dosage of 20 mg/kg revealed focal lobular infiltration by inflammatory cells (red arrows) (Figures 7F,G). Lastly, the liver sections of infected mice treated with statins at a dosage of 40 mg/kg demonstrated focal lobular infiltration by inflammatory cells (red arrows) and a few apoptotic figures (blue arrows) (Figures 7H,I).

### 3.3 Statins improved liver enzymes levels induced by *G. lamblia* infection

**Figure 8:**
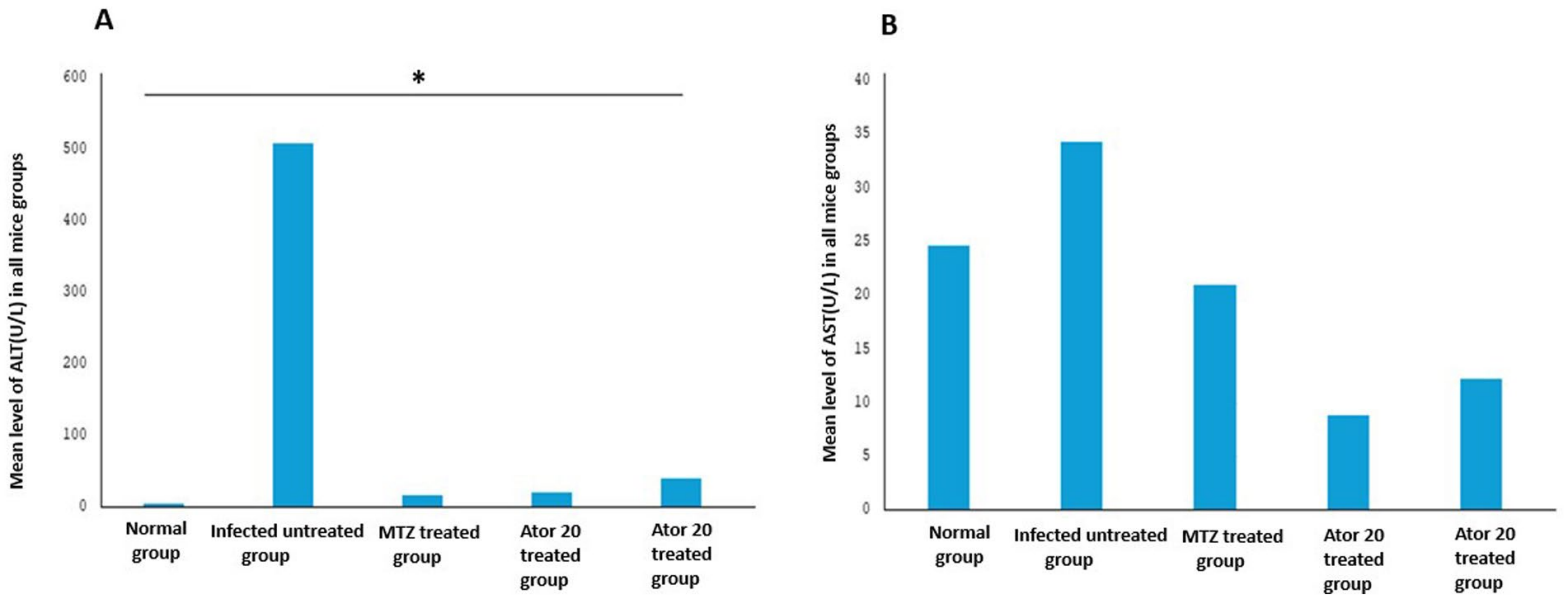
Effect of treatments on the mean ALT **(A)** and AST **(B)** level in all mice groups. Data are expressed as means (*n* = 10) with error bars representing SD and were analyzed using ANOVA with Tukey correction as a hoc test. Asterisk (*) indicates a significant difference in treated groups compared to the infected untreated group (*p* = 0.001).

Next, we measured the levels of serum ALT and AST as markers for liver function. Regarding ALT level, untreated mice showed significantly higher ALT level when compared to normal group (*p* = 0.001). Treatment of infected animals with either Atorvastatin or Metronidazole caused statistically significant reduction in ALT level in comparison to the untreated animals (*p* = 0.001, 0.002 and 0.004 for Atorvastatin 20 mg/kg and 40 mg/kg and Metronidazole treated groups respectively). Non-statistically significant difference was reported between Atorvastatin and Metronidazole -treated groups (*p* = 0.973 and 797 for Atorvastatin 20 mg/kg and 40 mg/kg versus Metronidazole treated animals, Figure 8A).

On the other hand, regarding AST level, our findings showed higher AST level in untreated mice when compared to normal group (with no statistical significance, *p* = 0.895). Treatments with Metronidazole (*p* = 0.803) or Atorvastatin 20 mg/kg (*p* = 0.289) or 40 mg/kg (*p* = 0.847) caused marked reduction in AST level when compared to untreated animals with no statistical significance. Moreover, non-statistically significant difference was recognized between animals treated with Atorvastatin either at a dose of 20 mg/kg (p = 0.847) or 40 mg/kg (*p* = 0.948) showed in comparison to Metronidazole -treated animals (Figure 8B).

### 3.4 Atorvastatin reduced the on IL6 expression in intestinal sections of infected mice

**Figure 9:**
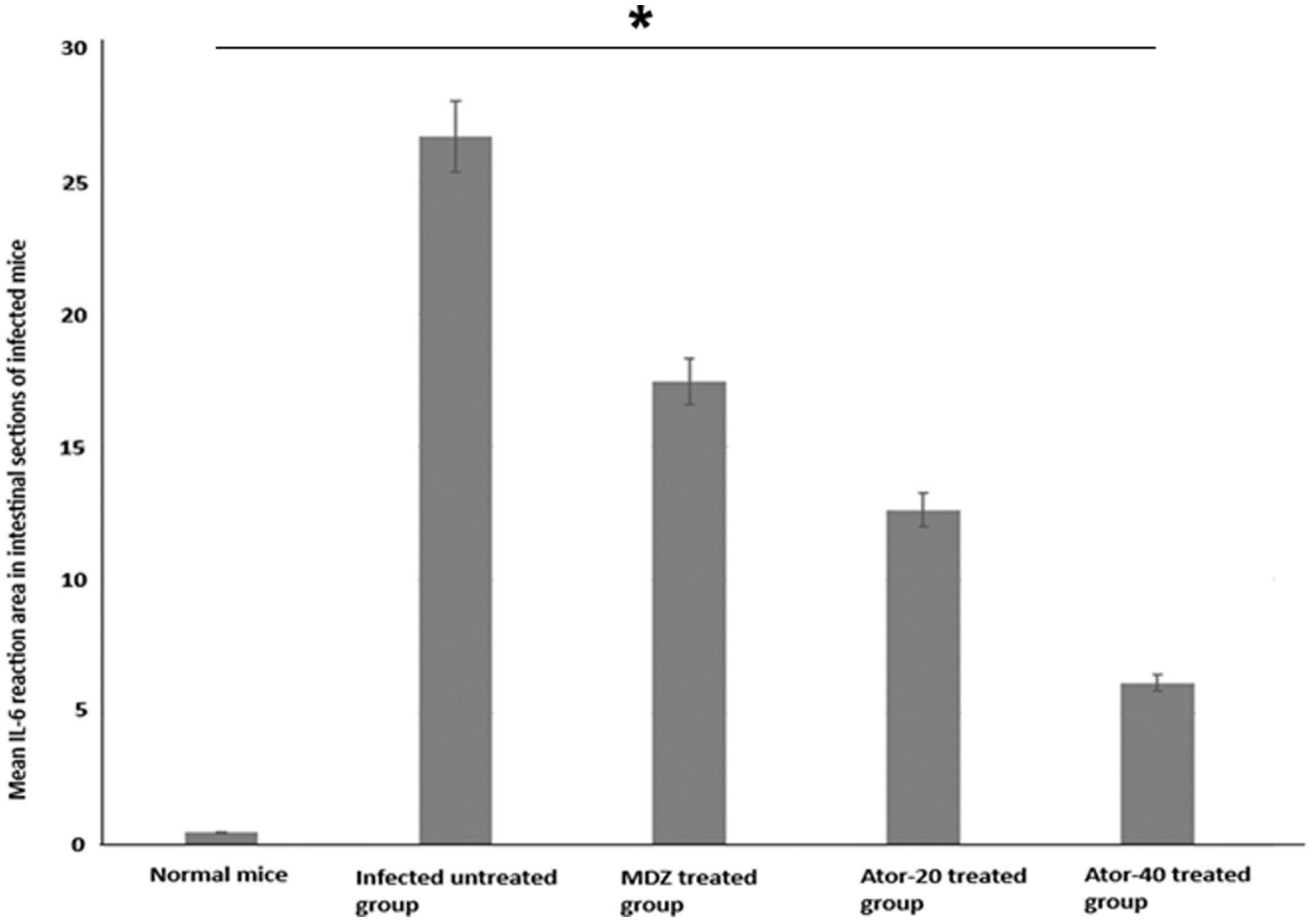
Illustrates the impact of treatments with Atorvastatin and Metronidazole on the expression of IL-6 in the intestinal sections of infected mice. The data are presented as means, with error bars indicating standard deviation, and were subjected to analysis via ANOVA with Tukey corrections for pairwise comparisons. Asterisk (*) indicates significant differences in the mean reaction area in all treated groups when compared to the infected untreated group.

**Figure 10:**
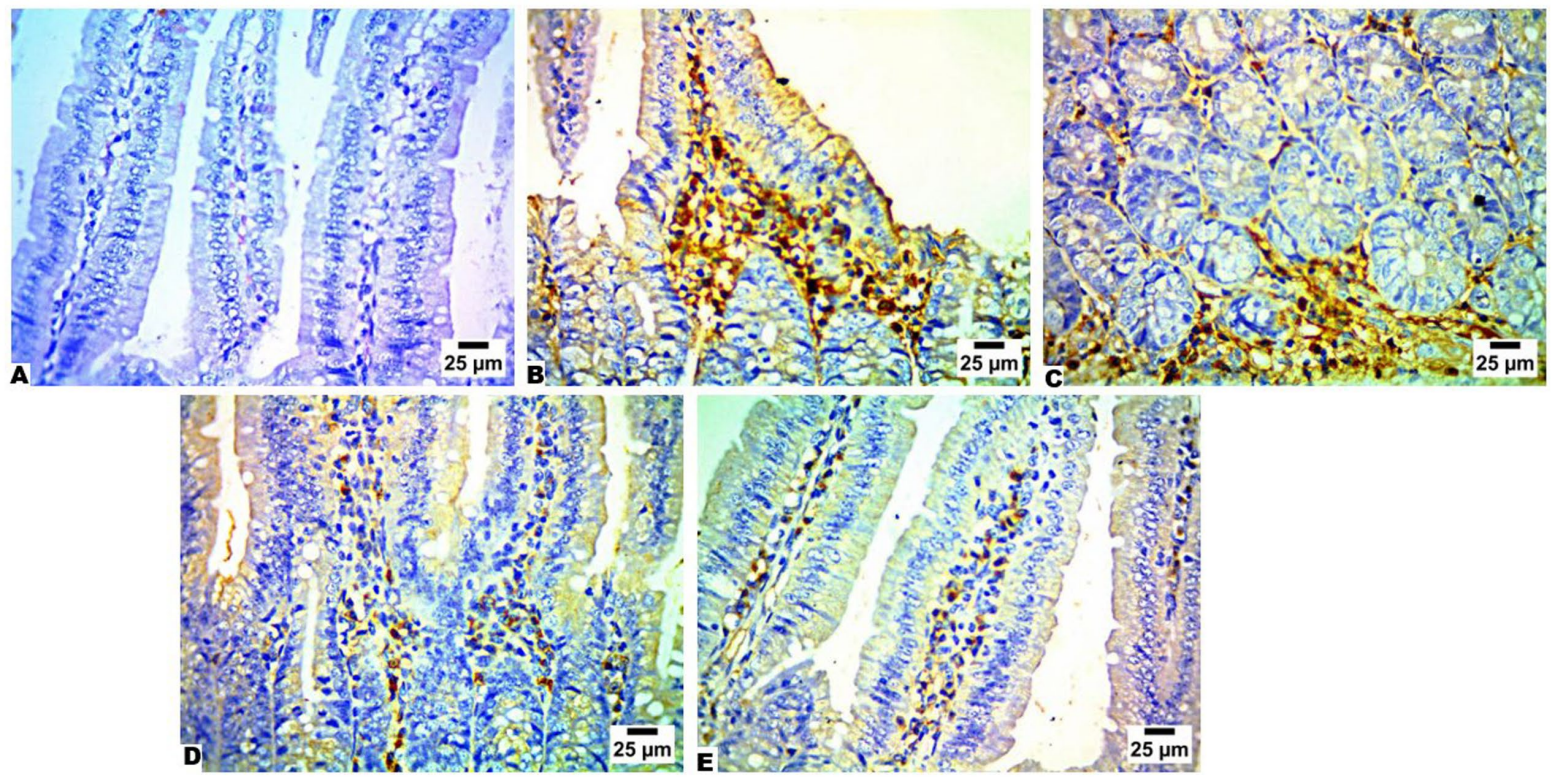
Photomicrograph showing the reaction area for IL 6 expression in intestinal sections of all animal groups (IHC-Peroxidase-DAB). **(A)** Sections from intestinal tissues of normal mice group showing negative expression for IL-6. **(B)** Sections from intestinal tissues of infected untreated animals showing severe positive expression for IL-6. **(C)** Sections from intestinal tissues from Metronidazole treated animals showing moderate positive expression for IL-6. **(D)** Sections from intestinal tissues from Atorvastatin-20 treated animals showing moderate positive expression of IL-6. **(E)** Sections from intestinal tissues from Atorvastatin-40 treated animals showing mild positive expression for IL-6.

The expression levels of the pro-inflammatory cytokine Interleukin-6 (IL-6) in the intestinal tissues of untreated mice infected with *Giardia* were significantly increased, with an average reaction area of 26.69% (showing a statistically significant difference when compared to normal mice, *p* = 0.001). This elevated level indicates the severity of the inflammatory response triggered by the parasite. Treatment with the standard anti-giardial medication Metronidazole led to a noticeable, albeit moderate, decrease in IL-6 expression, reducing the average reaction area to 17.48%. In contrast, the administration of Atorvastatin exhibited a superior, dose-dependent anti-inflammatory effect. The lower dose (Ator 20) was more effective in reducing IL-6 expression than Metronidazole, achieving an average of 12.63% (*p* = 0.002). Most notably, the higher dose of Atorvastatin (Ator 40) emerged as the most effective intervention, lowering IL-6 expression to a minimal average of 6.12% (*p* = 0.001). This signifies a substantial reduction compared to the untreated infected group and indicates that Atorvastatin, especially at the higher dosage, has a significant immunomodulatory effect that greatly alleviates the IL-6-mediated inflammatory cascade in the context of *G. lamblia* infection (Figures 9, 10).

### 3.5 Effect of statin treatment on iNOS expression in intestinal sections of infected mice

**Figure 11:**
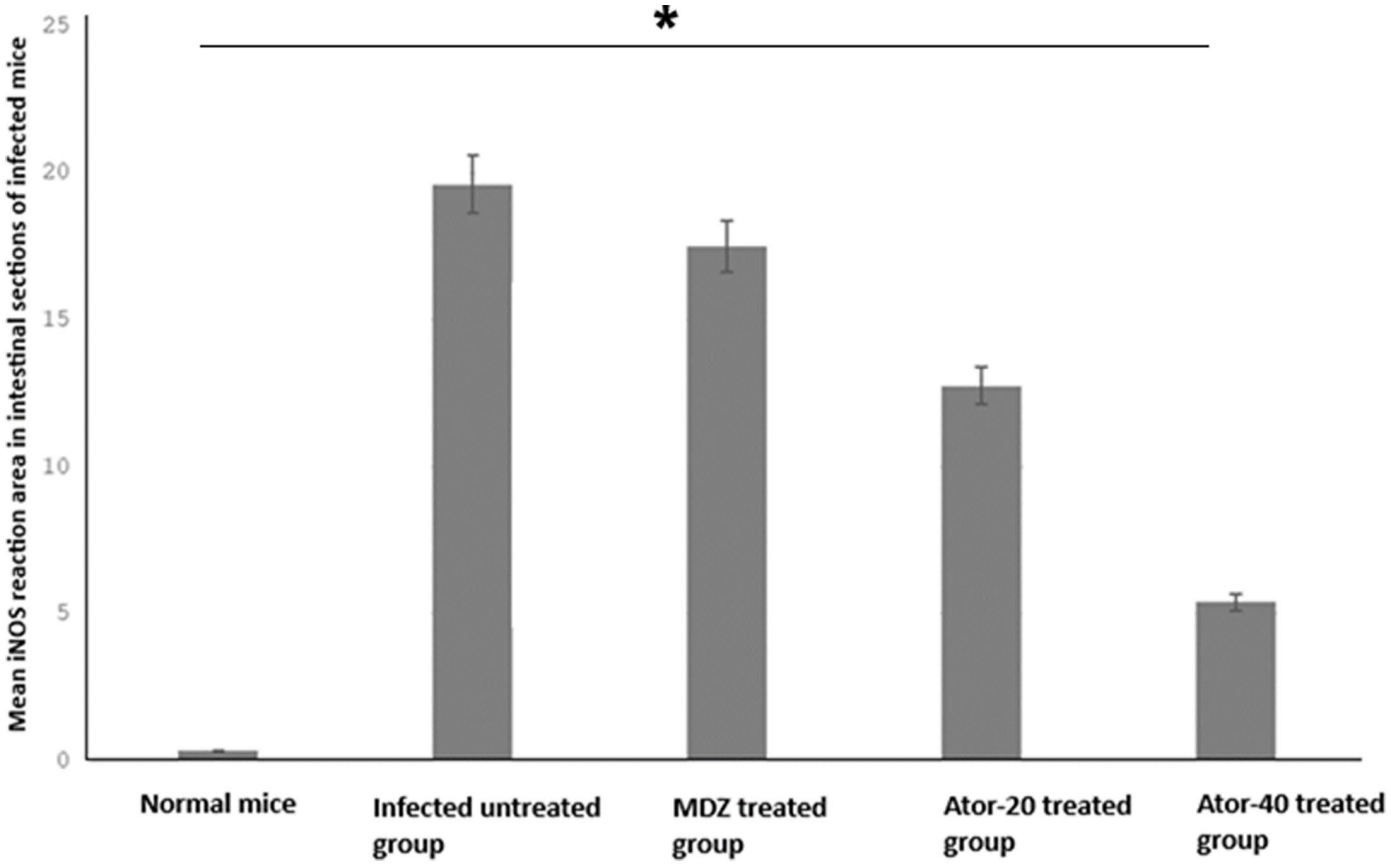
Illustrates the impact of treatments with statins and Metronidazole on the expression of iNOS in the intestinal sections of infected mice. Data are presented as means, with error bars indicating standard deviation, and were subjected to analysis via ANOVA with Tukey corrections for pairwise comparisons. Asterisk (*) indicates significant differences in the mean reaction area in all treated groups when compared to the infected untreated group.

**Figure 12:**
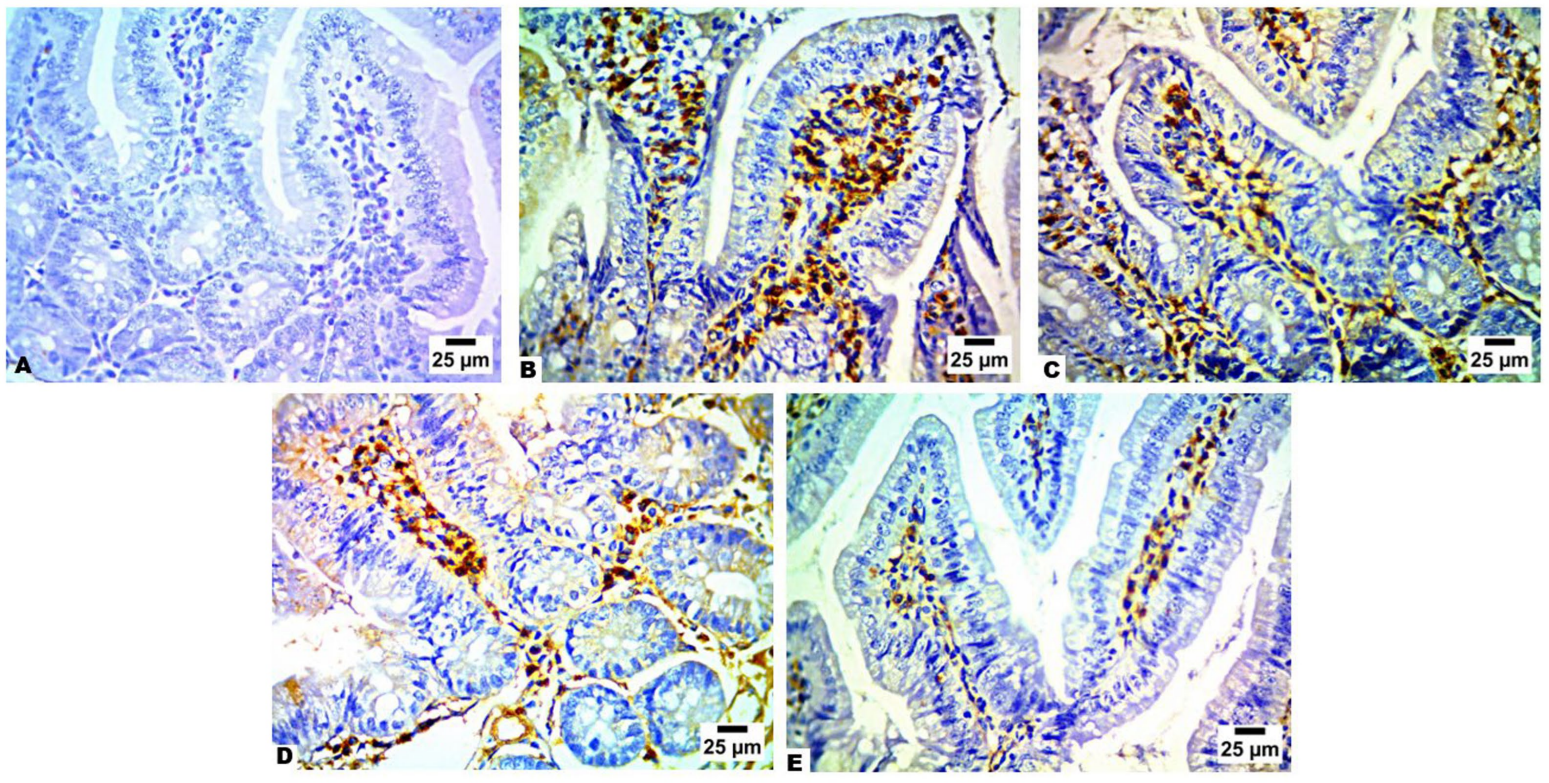
photomicrograph showing the reaction area for iNOS expression in intestinal sections of all animal groups (IHC-Peroxidase-DAB). **(A)** Sections from intestinal tissues of normal mice group showing negative expression for iNOS. **(B)** Sections from intestinal tissues of infected untreated animals showing severe positive expression for iNOS. **(C)** Sections from intestinal tissues from Metronidazole treated animals showing moderate positive expression for iNOS. **(D)** Sections from intestinal tissues from Atorvastatin-20 treated animals showing moderate positive expression of iNOS. **(E)** Sections from intestinal tissues from Atorvastatin-40 treated animals showing mild positive expression for iNOS.

The expression of inducible Nitric Oxide Synthase (iNOS), a crucial enzyme involved in the inflammatory pathway, reflected the pattern seen with IL-6. The Giardia-infected group that did not receive treatment exhibited a high average iNOS reaction area of 19.59% - in contrast to normal mice - confirming significant inflammatory activity (*p* = 0.001). Treatment with Metronidazole resulted in a slight reduction in iNOS expression to an average of 17.49%, indicating a limited impact on this particular inflammatory mediator (*p* = 0.028 when compared to the infected untreated mice group). In sharp contrast, the addition of Atorvastatin resulted in a significant and dose-dependent decrease in iNOS expression. The lower dose of Atorvastatin (20 mg/kg) reduced the iNOS reaction area to 12.75%, demonstrating a more pronounced anti-inflammatory effect than Metronidazole (*p* = 0.001 and 0.002 when compared to the infected untreated and Metronidazole -treated mice groups). The most significant effect was observed with the higher dose of Atorvastatin (40 mg/kg), which lowered iNOS expression to an exceptionally low average of 5.40% (*p* = 0.001, 0.001, and 0.002 when compared to the infected untreated, Metronidazole -treated, and Atorvastatin-20 mg/kg treated mice groups). This remarkable reduction, which is nearly four times lower than that of the untreated infected group, underscores the powerful anti-inflammatory and immunomodulatory effects of Atorvastatin in the intestinal tissue, indicating its potential to effectively mitigate the nitric oxide-mediated inflammatory damage caused by *G. lamblia* infection (Figures 11, 12).

## 4 Discussion

The present study investigated the therapeutic potential of Atorvastatin, a commonly prescribed HMG-CoA reductase inhibitor, as a novel treatment strategy for *G. lamblia* infection, both as a direct antiparasitic agent targeting the mevalonate pathway and as a modulator of the host’s inflammatory response. The findings integrate molecular docking simulations with comprehensive *in vivo* efficacy, histopathological, and immunomodulatory assessments in a murine model of giardiasis.

The initial molecular docking analysis provided a compelling rationale for testing Atorvastatin against *G. lamblia*. The study targeted four key enzymes in the mevalonate pathway of the parasite: farnesyl transferase (ftase), isopentenyl pyrophosphate isomerase (ipp), mevalonate diphosphate decarboxylase (mvd), and mevalonate kinase (mvk). This pathway is crucial for the parasite’s survival, as it is involved in the synthesis of essential isoprenoids. As shown in the present study, across the four targets, Atorvastatin exhibited slightly more favorable docking energies, particularly for IPP and MVD, and often utilizes a combination of hydrogen bonding and hydrophobic interactions. This suggests that Atorvastatin may exert its anti-giardial effect by interfering with the parasite’s mevalonate pathway, a mechanism distinct from the DNA-damaging action of Metronidazole. This molecular evidence supports the hypothesis that statins can act as repurposed drugs targeting essential metabolic processes in *G. lamblia* (20).

The in vivo results confirmed that Atorvastatin possesses significant antiparasitic activity, leading to a dose-dependent reduction in trophozoite counts in the small intestine of infected mice. The higher dose of Atorvastatin (40 mg/kg) achieved a 41.54% reduction, while the lower dose (20 mg/kg) resulted in a 27.30% reduction (*p* = 0.002 and *p* = 0.004, respectively when compared to infected untreated mice). This aligns with previous research indicating the anti-parasitic properties of statins against various protozoan infections, including *Cryptosporidium* spp., *Trypanosoma cruzi, Lieshmania* spp. and *Plasmodium* spp. (15–17, 40). The mechanism, likely involving the disruption of isoprenoid synthesis, could synergize with other anti-parasitic agents. The observed reduction in trophozoite counts by Atorvastatin can be attributed to its interference with the mevalonate pathway in *Giardia*. *G. lamblia* relies on this pathway for the synthesis of essential isoprenoids, which are crucial for cellular functions such as protein isoprenylation, membrane integrity, and growth (14, 41).

Furthermore, our study revealed that Atorvastatin significantly improved liver function parameters, specifically reducing elevated serum ALT and AST levels caused by *G. lamblia* infection. This protective effect on the liver is a crucial finding, as giardiasis is known to induce hepatic pathology, including steatosis and inflammatory lesions (42, 43). The ability of statins to improve liver function in the context of parasitic infections is consistent with their established hepatoprotective effects, which extend beyond their lipid-lowering capabilities to include anti-inflammatory and antioxidant properties (44–48). This suggests that Atorvastatin not only targets the parasite but also helps to mitigate the host’s inflammatory response and liver damage induced by the infection.

In terms of intestinal pathology, statin treatment resulted in a mostly regular villous pattern with mild chronic inflammation, indicating a restorative effect on the intestinal architecture. The observed improvement in intestinal morphology may be attributed partially to reduced trophozoite count and reduced expression of the inflammatory markers interleukin-6 (IL-6) and inducible nitric oxide synthase (iNOS) in the intestinal tissue, which demonstrates a significant therapeutic advantage of statin treatment over Metronidazole in mice infected with *G. lamblia*. This finding provide critical insight into the immunomodulatory potential of statins as an adjunctive therapy for giardiasis, a parasitic infection characterized by significant host inflammatory responses.

Elevated IL-6 levels are a characteristic feature of giardiasis in both human patients and mouse models (49–51). While IL-6 is crucial for the effective clearance of the parasite (52), its over-expression contributes to the pro-inflammatory state that drives intestinal damage (50). The parasite can trigger the release of pro-inflammatory cytokines, notably IL-6, by activating specific signaling pathways such as p38 and ERK (53). Consequently, the elevated levels of IL-6 seen in both the untreated and Metronidazole-treated groups likely indicate the persistence of a damaging inflammatory response initiated by the parasite. The study indicates that while IL-6 level in statin-treated animals were reduced, it remained slightly elevated compared to those in the untreated infected group. This is concerning, as lower IL-6 level is linked to increased vulnerability to infections due to diminished immune response and impaired inflammatory processes. Nonetheless, statins managed to lower IL-6 levels enough to mitigate its potential pathological effects without causing significant immunosuppression drawbacks (54).

Inducible Nitric Oxide Synthase (iNOS) is responsible for the massive production of nitric oxide (NO), a potent free radical, in response to pro-inflammatory stimuli. In the context of intestinal infection, iNOS is upregulated in intestinal epithelial cells (IECs) and macrophages (55, 56). While NO is an important component of the host’s defense mechanism against pathogens, excessive and sustained NO production, driven by iNOS, can lead to cellular damage, oxidative stress, and impaired barrier function in the intestinal epithelium (57). The reduction in iNOS expression in our statin-treated group suggests a successful dampening of this destructive inflammatory loop, which is consistent with the observed preservation of intestinal tissue integrity.

The superior therapeutic efficacy of statins in reducing IL-6 and iNOS expression is attributable to their well-documented pleiotropic effects, which extend beyond their primary role in cholesterol reduction (58). This inhibition prevents the prenylation of small GTPases (like Rho and Rac), which are essential for the activation of key inflammatory transcription factors, most notably Nuclear Factor-kappa B (NF-κB) (59). The suppression of the NF-κB pathway is directly linked to the downregulation of both IL-6 and iNOS. Multiple studies have confirmed that statins can significantly reduce the production of pro-inflammatory cytokines, including IL-6, in various cell types (60, 61). Furthermore, statins have been shown to attenuate inflammatory responses by suppressing the TLR4/MyD88/NF-κB axis, which directly results in lower iNOS expression (62).

## 5 Conclusion

Atorvastatin emerges as proof-of-concept for repurposed statins in veterinary giardiasis, uniquely combining metabolic disruption, anti-inflammatory modulation, and organ protection. These preclinical data justify controlled field trials in dogs/cats, resistance monitoring, and combination regimens to combat a neglected zoonosis.

### 5.1 Study limitations

Reliance on male C57BL/6 mice introduces sex bias; females exhibit heightened Th2/IL-6 responses that may alter efficacy, warranting inclusion in Phase II studies. H&E trophozoite counts, while standard, lack cyst-stage specificity and genomic confirmation—future qPCR for gdh/triose phosphate isomerase loci and *Giardia*-IHC will quantify burdens precisely. *In vitro* axenized trophozoite/cyst assays with radiolabeled mevalonate precursors will dissect direct vs. host effects. Short-term outcomes preclude chronic clearance/recurrence assessment.

## Data Availability

The datasets presented in this study can be found in online repositories. The names of the repository/repositories and accession number(s) can be found in the article/supplementary material.
